# Endovascular repair of infrarenal aortic aneurysm and severe stenosis in Type V Takayasu arteritis: a rare case report and clinical insights

**DOI:** 10.1186/s43044-025-00702-7

**Published:** 2025-12-03

**Authors:** Much. Muzakky Misbachul Firdaus, Zakiyyatul Aflakha, Yusuf Aji Samudera Nurrobi, Johannes Nugroho Eko Putranto

**Affiliations:** 1 Department of Cardiology and Vascular Medicine, Faculty of Medicine, Universitas Airlangga, Surabaya, East Java Indonesia; 2 Department of Cardiology and Vascular Medicine, Dr Soetomo General Academic Hospital, Surabaya, East Java Indonesia

**Keywords:** Takayasu arteritis, Limb ischemia, Endovascular aneurysm repair, EVAR, Aneurysm

## Abstract

**Background:**

Takayasu arteritis (TA) is a rare, chronic vasculitis affecting the aorta and its major branches, leading to stenosis, aneurysm, and ischemic complications. It primarily affects young adults and may progress to critical ischemia, necessitating revascularization strategies. Endovascular aneurysm repair (EVAR) is increasingly recognized as an effective, minimally invasive intervention for managing TA-associated complications.

**Case description:**

A 28-year-old male presented with a three-month history of progressive bilateral lower limb discomfort and tingling exacerbated by activity, intermittent abdominal cramps, and fatigue. Physical examination revealed a significant blood pressure discrepancy between the upper limbs (right: 193/66 mmHg, left: 100/71 mmHg), reduced lower limb pressures (right ankle: 66/41 mmHg, left ankle: 72/42 mmHg), pulse deficits, and diminished ankle-brachial indices (right: 0.34; left: 0.37). Transthoracic echocardiography (TTE) demonstrated all-chamber dilatation, reduced systolic function (EF: 38%), severe mitral regurgitation, moderate aortic regurgitation, and a large right atrial thrombus (3.0 × 1.9 cm). Computed tomography angiography (CTA) revealed critical mid-aortic stenosis (6.9 × 11.9 mm), a post-stenotic saccular aneurysm (20.6 × 22.4 mm), and severe narrowing of the left subclavian and axillary arteries. The patient underwent endovascular aneurysm repair (EVAR), with a thoracic stent graft (24 × 80 mm) successfully deployed. Six months post-procedure, the patient experienced complete resolution of symptoms, with improved ABI values (right: 0.94; left: 0.73) and significant regression of the aneurysm (13.1 × 13.1 mm).

**Conclusion:**

EVAR may represent a feasible option for carefully selected TA patients with focal lesions and high surgical risk, although open repair remains necessary for more complex disease. Lifelong surveillance and further research are essential to optimize management strategies in this rare condition.

## Background

Takayasu arteritis (TA) is a rare, chronic granulomatous large-vessel vasculitis primarily affecting the aorta and its major branches, with a strong predominance among young adults, especially females [1, 2]. A recent systematic review estimated the global incidence of TA to be approximately 1.11 cases per million person-years [3]. Epidemiological studies consistently show a markedly higher incidence of TA in Asian populations compared to Europe and North America, with estimates in Asia often reported to be up to 100 times greater than in Western countries [4]. To date, no specific epidemiological data on TA is available for Indonesia, underscoring a gap in regional understanding of this condition.

TA typically progresses through two distinct phases: an acute inflammatory phase and a chronic vascular phase [5, 6]. During the initial inflammatory phase (also termed the “pre-pulseless” stage), patients frequently exhibit nonspecific constitutional symptoms, including fever, fatigue, arthralgia, and weight loss [7, 8]. As disease progresses into the chronic phase (often referred to as the “pulseless” stage), persistent granulomatous inflammation drives intimal proliferation, arterial wall thickening, and remodeling, culminating in stenosis, occlusion, or aneurysmal dilation [5, 8]. In this context, we report a rare and challenging case of TA complicated by severe infrarenal abdominal aortic stenosis and concomitant saccular aneurysm, successfully managed with EVAR strategy.

## Case presentation

A 28-year-old male presented with a three-month history of bilateral lower limb discomfort, tingling sensations, and intermittent swelling, worsening during physical activity and relieved with rest, consistent with intermittent claudication. The claudication was sufficiently severe to limit his ability to walk long distances and significantly impaired his daily activities. Additional symptoms included intermittent abdominal cramps, generalized fatigue, and swelling of the lower limbs when the patient was in a prolonged lying position. The patient denied a history of smoking, cardiovascular disease, autoimmune disorders, or previous vascular intervention.

**Fig. 1:**
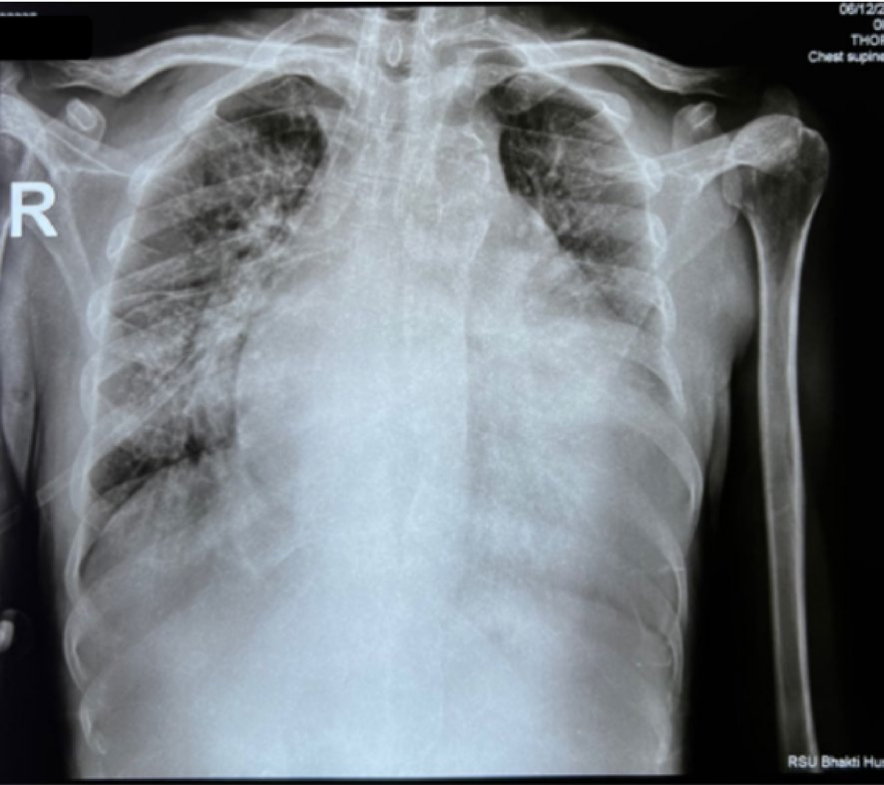
Chest X-ray demonstrating significant cardiomegaly, characterized by an enlarged cardiac silhouette, suggesting advanced cardiac involvement

On physical examination, there was a marked discrepancy in blood pressure between the upper limbs (right arm 193/66 mmHg vs. left arm 100/71 mmHg) and markedly reduced pressures in the lower extremities (right ankle 66/41 mmHg, ABI 0.34; left ankle 72/42 mmHg, ABI 0.37). Bilateral lower limb pulse deficits were present. Cardiovascular auscultation revealed an apical systolic murmur and a diastolic murmur at the second left intercostal space. A prominent abdominal aortic bruit was also audible. The heart rate was irregular at 61 beats per minute, the respiratory rate 18 breaths per minute, and body temperature 36.7 °C. ECG demonstrated atrial fibrillation with a moderate ventricular response and features of left ventricular hypertrophy, while chest radiography showed cardiomegaly (Fig. 1). Inflammatory markers demonstrated an elevated C-reactive protein (CRP) level of 0.55 mg/dL and an increased erythrocyte sedimentation rate (ESR) of 44 mm/h.

Transthoracic echocardiography (TTE) revealed biventricular dilatation with severe left ventricular systolic dysfunction (EF 38%) and Grade III diastolic dysfunction, consistent with advanced myocardial involvement. There was severe mitral regurgitation from anterior leaflet prolapse and moderate aortic regurgitation, both contributing to volume overload. A large right atrial thrombus (3.0 × 1.9 cm) was also visualized. Pulmonary pressures were elevated, indicating secondary pulmonary hypertension.

**Fig. 2:**
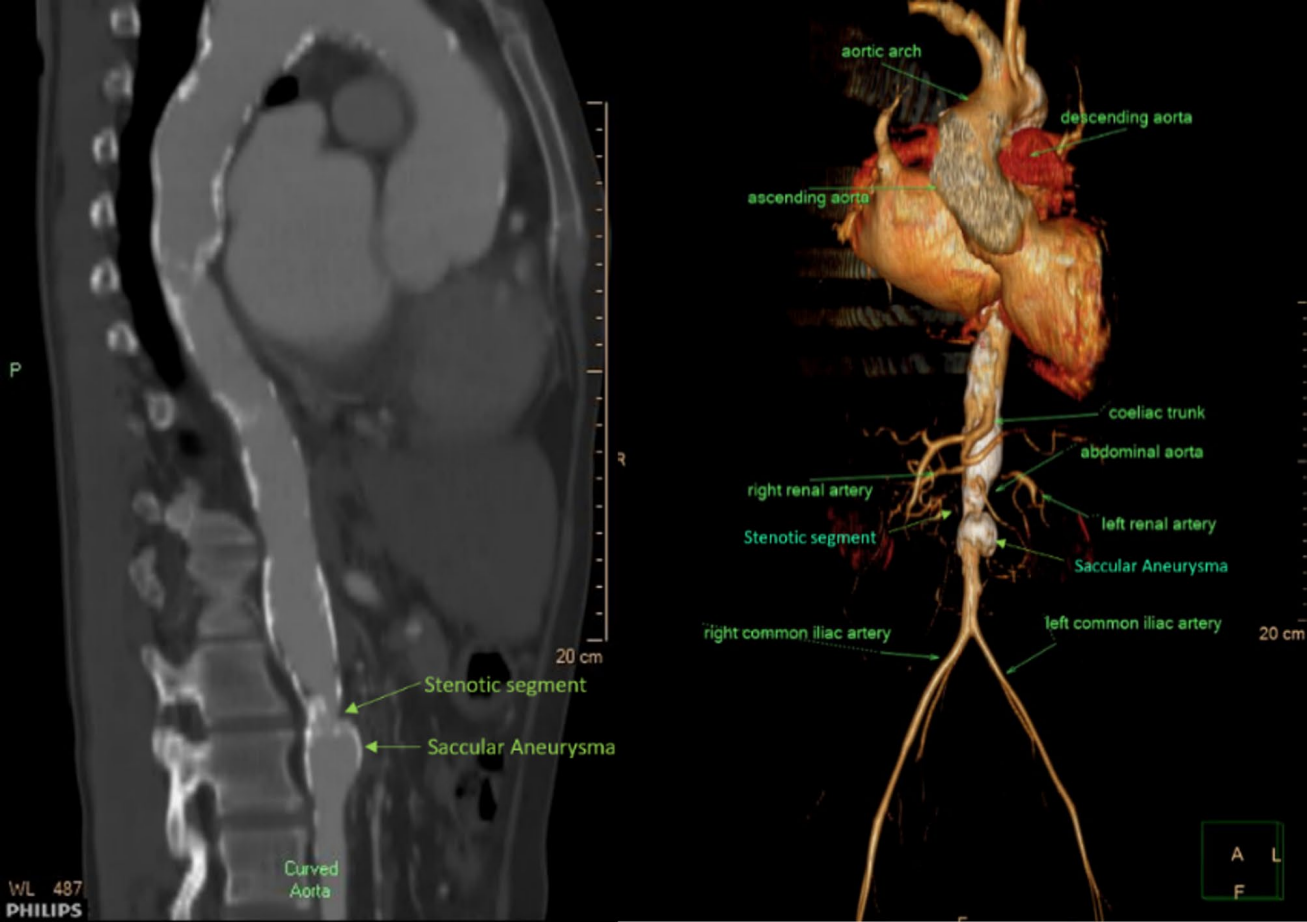
Computed tomography angiography (CTA) revealing significant stenosis in the infrarenal abdominal aorta accompanied by a saccular aortic aneurysm

CT angiography (CTA) of the thoracoabdominal aorta demonstrated extensive calcified plaques extending from the thoracic through the abdominal segments. A critical stenosis was identified in the infrarenal abdominal aorta, measuring approximately 6.9 × 11.9 mm, associated with a post-stenotic saccular aneurysm measuring 20.6 × 22.4 mm (Fig. 2). Previous CTA of the upper extremities revealed a diffusely narrowed left subclavian artery (~ 4 mm) and left axillary artery (~ 4 mm). Based on the angiographic findings, the case was classified as Type V Takayasu arteritis according to Numano’s classification, which involves the thoracic aorta, abdominal aorta, and the branches of aortic arch.

**Fig. 3:**
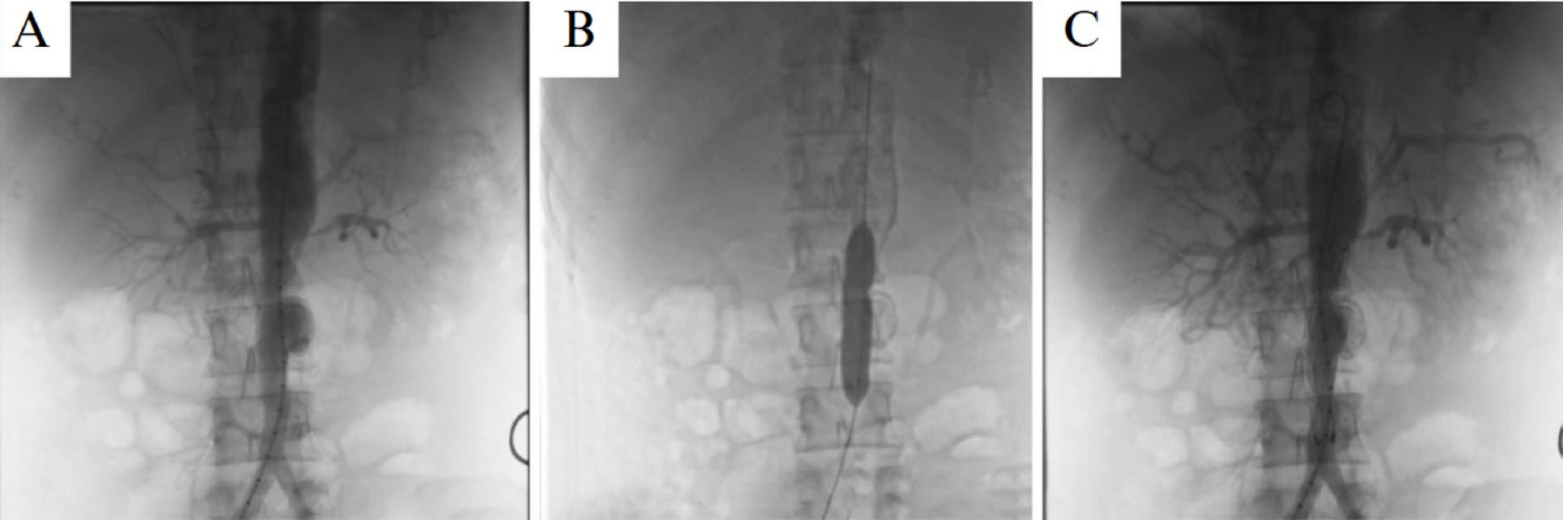
Sequential aortographic images depicting the endovascular intervention process. **A** The pre-stent image shows critical stenosis in the infrarenal abdominal aorta and the associated aneurysmal dilation. **B** The mid-procedure image illustrates balloon pre-dilation of the stenotic segment to prepare for stent deployment. **C** The post-stent image demonstrates successful restoration of luminal patency with complete exclusion of the aneurysmal sac

**Fig. 4:**
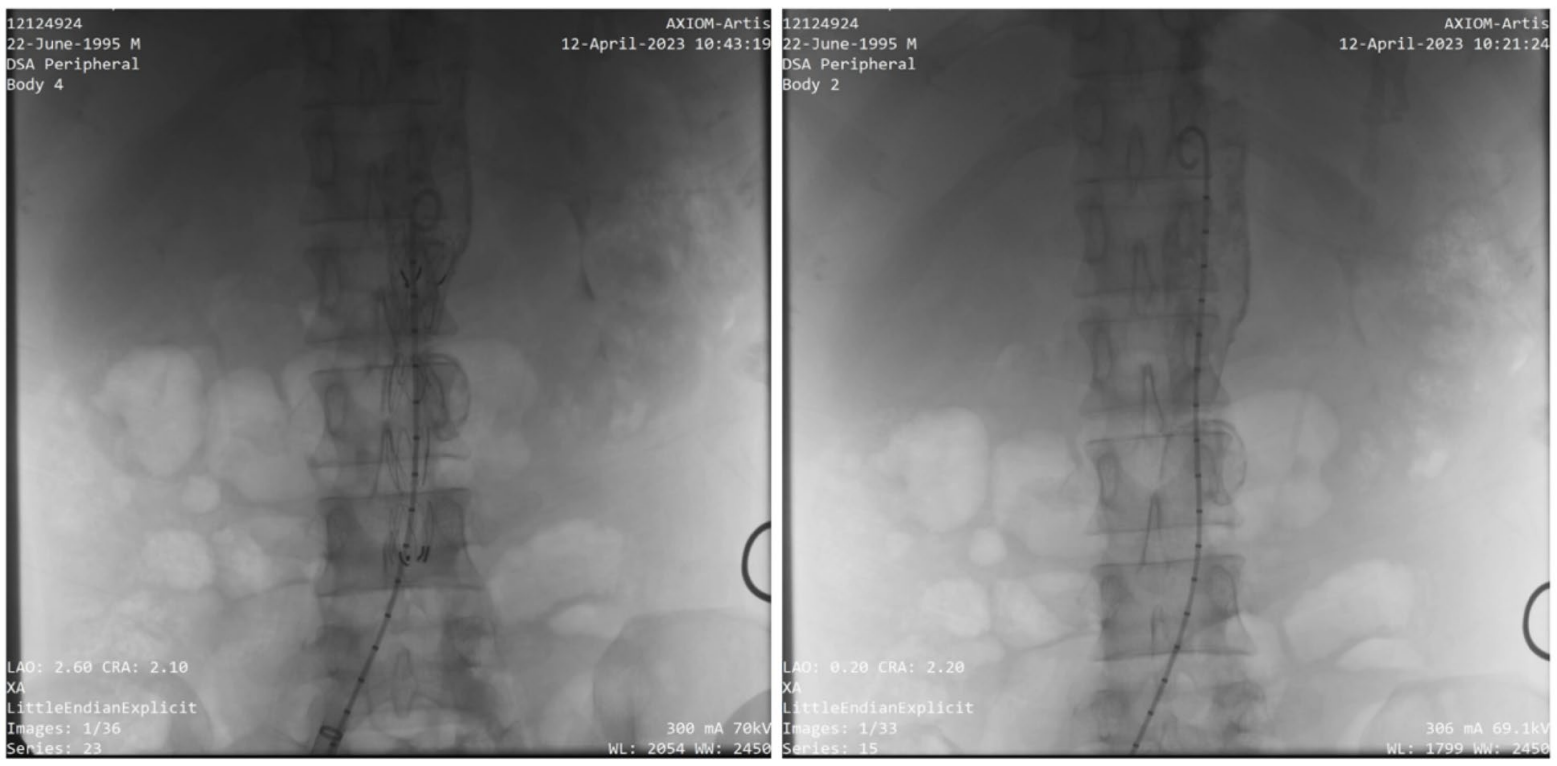
Pre- and post-procedure aortographic images illustrating the successful placement of the Expandable Seal Thoracic Stent Graft (24 × 80 mm)

Following a multidisciplinary team (MDT) discussion involving interventional cardiologist and rheumatologist, the patient underwent endovascular aneurysm repair (EVAR) as the preferred strategy, given the high surgical risk associated with his cardiac comorbidities. A right femoral artery catheterization was performed using the Seldinger technique percutaneously. Following successful arterial access, systemic anticoagulation was achieved with an intravenous bolus of 5,000 units of unfractionated heparin to prevent procedure-related thrombosis. An 8 F sheath was initially placed, followed by insertion of a 5 F JR 4.0 catheter to perform aortography. The imaging confirmed critical mid-aortic stenosis with no renal artery involvement and the saccular aneurysm just distal to the stenotic region (Fig. 3). The 8 F sheath was subsequently exchanged for an 18 F sheath, and the stenotic segment was pre-dilated using Armada 35 balloons (10 × 80 mm and 14 × 60 mm; Abbott Vascular, Santa Clara, CA, USA). A stiff guidewire was advanced and an Expandable SEAL Thoracic Stent Graft (24 × 80 mm; S&G Biotech Co., Ltd., Seoul, South Korea) was deployed to exclude the saccular aneurysm and restore aortic patency (Fig. 4). Self-expandable graft was preferred due to its greater conformability, reduced risk of vessel trauma in the stenotic/aneurysmal segment, and adaptability to dynamic changes. Post-procedural angiography demonstrated excellent stent positioning, restored aortic patency, and complete exclusion of the aneurysm without evidence of endoleak.

**Fig. 5:**
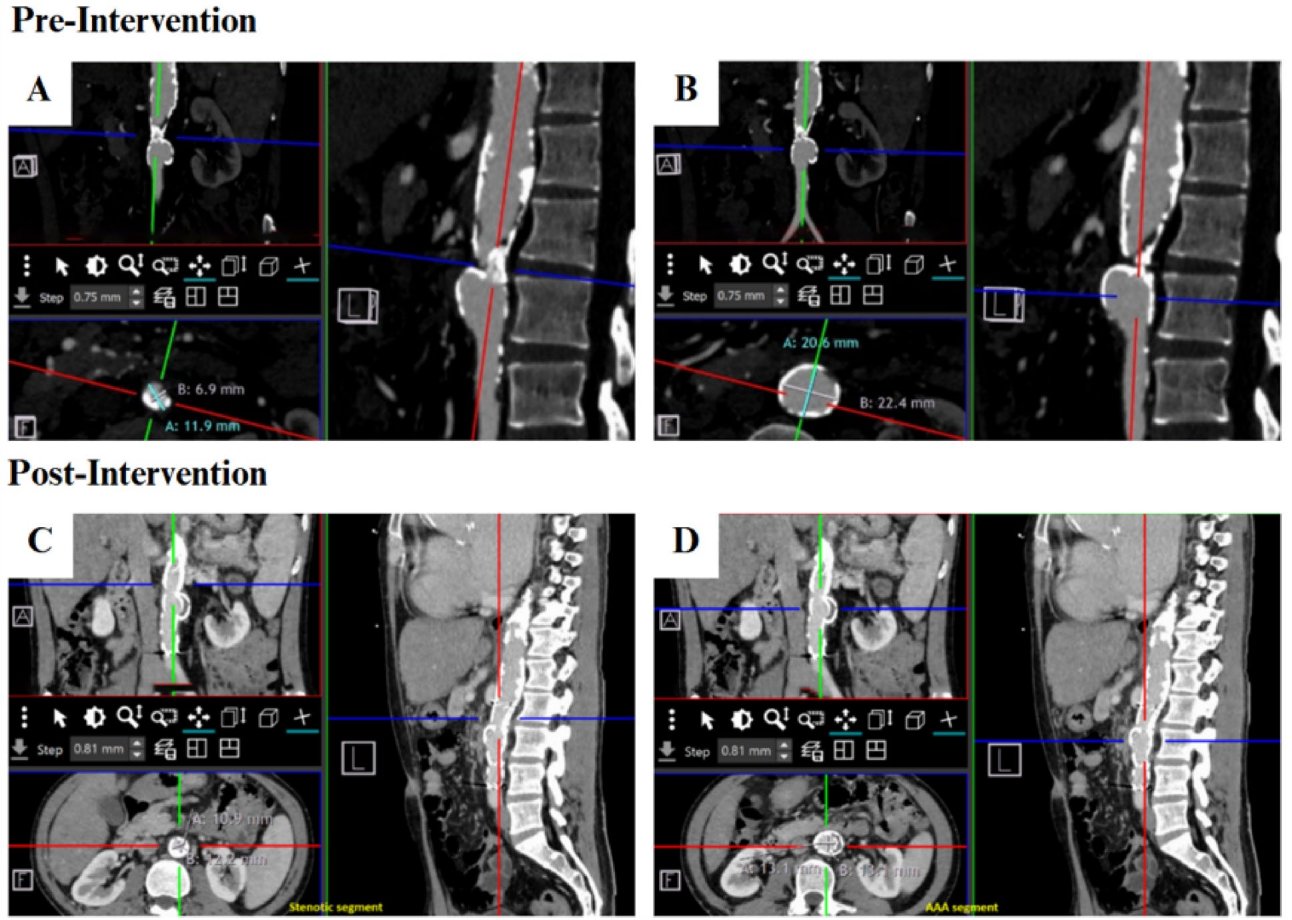
Six-month postoperative thoracoabdominal CTA. Post-endovascular repair, the stenotic infrarenal aorta improved from 6.9 × 11.9 mm to 10.9 × 12.2 mm. The saccular abdominal aortic aneurysm decreased from 20.6 × 22.4 mm to 13.1 × 13.1 mm

The patient recovered without complications and was discharged on clopidogrel 75 mg o.d., warfarin 2 mg o.d., atorvastatin 40 mg o.d., heart failure medications, together with high-dose oral methylprednisolone 1 mg/kg/day. No adjunctive steroid-sparing agents such as methotrexate, azathioprine, or biologics were administered. At six months, the patient reported complete resolution of bilateral lower-limb pain and swelling, with markedly improved exercise tolerance and full restoration of ambulatory capacity, reflecting successful reversal of previously debilitating claudication. Physical examination showed right arm blood pressure of 189/70 mmHg, left arm 94/65 mmHg, right ankle 178/67 mmHg (ABI 0.94), and left ankle 138/66 mmHg (ABI 0.73). Repeat thoracoabdominal CTA confirmed excellent stent patency without endoleak. The previously stenotic segment had improved to 10.9 × 12.2 mm, while the aneurysmal segment showed significant regression to 13.1 × 13.1 mm (Fig. 5). Recognizing the potential for late complications in Takayasu arteritis—such as restenosis, graft occlusion, or disease relapse—lifelong clinical and imaging surveillance is essential to ensure long-term EVAR durability and sustained disease control. The patient’s ongoing management will include multidisciplinary follow-up involving cardiology, cardiovascular surgery, and rheumatology teams, with CTA surveillance every six months to monitor stent integrity and disease progression. Immunosuppressive therapy will be continuously optimized to maintain remission, and periodic multidisciplinary reassessment will guide the appropriate timing of any future cardiac interventions, including potential valvular repair or replacement once systemic stability is achieved.

## Discussion

The present case represents a rare manifestation of TA and expands the spectrum of endovascular management in this disease. Although aneurysmal involvement of the aorta in TA has been described sporadically, infrarenal aneurysms are uncommon. Prior literature document infrarenal aortic aneurysms and isolated reports of concomitant stenosis and aneurysmal dilatation [9]. Endovascular aortic repair has been reported in TA patients, but predominantly in the context of extensive thoracoabdominal disease requiring complex chimney/sandwich techniques or hybrid approaches using direct aortic access for device deployment [10, 11]. To our knowledge, there are no prior peer-reviewed reports describing the use of standard EVAR to treat a short-segment infrarenal stenosis immediately followed by a post-stenotic saccular aneurysm in TA. Furthermore, the clinical scenario was uniquely challenging due to the coexistence of multiple comorbidities, including heart failure with reduced ejection fraction, atrial fibrillation with right atrial thrombus, and significant valvular disease, all of which markedly increased the surgical risk and reinforced the rationale for an endovascular approach.

Early identification and treatment initiation remain important challenges in the treatment of TA. The disease’s rarity and the non-specific nature of the presenting symptoms often lead to a delayed diagnosis and late presentation. In this case, diagnosis was established using the American College of Rheumatology (ACR)/EULAR 2022 classification criteria for Takayasu arteritis, which demonstrate improved diagnostic accuracy compared to prior criteria, with reported sensitivity of 93% and specificity of 99% for TA [12]. The patient fulfilled several positive criteria, including leg claudication, an abdominal aortic bruit, systolic blood pressure differences between the arms, and involvement of three arterial territories (Table 1).

**Table 1:** ACR/EULAR 2022 classification criteria for Takayasu arteritis in the present case

Category	Specific criterion met	Patient finding	Score
Absolute requirements	Age ≤ 60 years at diagnosis	34-year-old male	Required
	Evidence of vasculitis on imaging	CT angiography: stenosis and aneurysm involving abdominal aorta	Required
Additional clinical criteria	Arm or leg claudication	Exertional leg pain with reduced ankle pressures	+ 2
	Vascular bruit	Abdominal aortic bruit audible on auscultation	+ 2
	Systolic BP difference ≥ 20 mmHg between arms	Right arm 193/66 mmHg vs. left arm 100/71 mmHg	+ 1
Additional imaging criteria	Three arterial territories involved (thoracic aorta, abdominal aorta, and aortic arch branch)	CT angiography confirmed lesions in thoracic aorta, abdominal aorta, and aortic arch branch	+ 3
Total score			8

^a^According to 2022 ACR/EULAR criteria, a score ≥ 5 confirms classification as Takayasu arteritis, provided absolute requirements are met.

The management of Takayasu arteritis encompasses medical therapy, endovascular procedures, and surgical vascular reconstruction, with the choice of treatment tailored to the patient’s specific clinical condition and disease stage [13]. During the active inflammatory phase, treatment primarily involves corticosteroids and immunosuppressive agents to mitigate both systemic and vascular inflammation. Revascularization in TA is indicated for anatomically or hemodynamically significant stenoses, progressive aneurysmal disease, or critical limb-threatening ischemia—ideally during a quiescent phase to optimize outcome and reduce the risk of restenosis and perioperative complications [14]. In the present case, the coexistence of critical infrarenal abdominal aortic stenosis endangering limb viability and coexisting saccular aneurysm defined a dual-risk scenario—ischemic compromise and potential rupture—that clearly justified urgent intervention.

To date, there are no guidelines that clearly mention the indications for choosing between endovascular versus open surgical revascularization. Therefore, revascularization strategy must be individualized, weighing lesion complexity, procedural risk, and patient comorbidities. Meta-analytic evidence demonstrates that endovascular interventions are associated with a significantly increased risk of restenosis compared with surgical approaches (OR 5.18; *p* < 0.001). Conversely, endovascular strategies confer a reduced risk of stroke (OR 0.33; *p* < 0.03). Mortality outcomes did not differ significantly between the two modalities (OR 0.84; *p* = 0.67) [15]. For focal lesions in patients with elevated surgical risk, endovascular options generally offer decreased perioperative morbidity and shorter recovery [15]. In this case, the combination of focal infrarenal disease requiring both limb salvage and aneurysm exclusion, patient preference, together with patient’s multiple comorbidities favoured EVAR strategy over open surgery in this case. Regardless of modality, optimal outcomes demand meticulous peri-procedural immunosuppression and vigilant long-term surveillance for restenosis or disease progression [14, 15]. 

## Conclusion

In summary, the diagnosis of TA remains challenging and necessitates a high level of clinical suspicion to ensure timely recognition and management. There are currently no universally accepted guidelines for choosing between endovascular repair and surgical intervention, requiring an individualized approach that considers lesion morphology, patient comorbidities, and surgical risk. EVAR is most suitable for patients with focal or limited arterial lesions who also have significant comorbidities or high perioperative risk, while open surgery should be reserved for those with extensive or complex arterial involvement. Long-term follow-up, including repeat imaging and clinical assessment, is essential to monitor disease progression and intervention outcomes. This case underscores the urgent need for more epidemiological and interventional studies focusing on Takayasu arteritis in Indonesia to inform management strategies and improve patient outcomes.

## Data Availability

No datasets were generated or analysed during the current study.

## Abbreviations

TA: Takayasu arteritis
EVAR: Endovascular aneurysm repair
ABI: Ankle-brachial index
LVH: Left ventricular hypertrophy
LVIDd: Left ventricular internal diameter in diastole
LVMI: Left ventricular mass index
RVDB: Right ventricular diastolic base diameter
TR Vmax: Tricuspid regurgitation maximum velocity
PVAccT: Pulmonary valve acceleration time
ERO: Effective regurgitant orifice
